# Polyphenols as Immunomodulators and Epigenetic Modulators: An Analysis of Their Role in the Treatment and Prevention of Breast Cancer

**DOI:** 10.3390/nu16234143

**Published:** 2024-11-29

**Authors:** Esmanur Eren, Jyotirmoyee Das, Trygve O. Tollefsbol

**Affiliations:** 1 Department of Biology, University of Alabama at Birmingham, Birmingham, AL 35294, USA; eeren@uab.edu (E.E.); jdas@uab.edu (J.D.); 2 Integrative Center for Aging Research, University of Alabama at Birmingham, Birmingham, AL 35294, USA; 3 O’Neal Comprehensive Cancer Research, University of Alabama at Birmingham, Birmingham, AL 35294, USA; 4 Nutrition Obesity Research Center, University of Alabama at Birmingham, Birmingham, AL 35294, USA; 5 Comprehensive Diabetes Center, University of Alabama at Birmingham, Birmingham, AL 35294, USA

**Keywords:** dietary polyphenols, breast cancer, immunomodulation, epigenetic

## Abstract

Breast cancer poses a substantial health challenge for women globally. Recently, there has been a notable increase in scholarly attention regarding polyphenols, primarily attributed to not only the adverse effects associated with conventional treatments but also their immune-preventive impacts. Polyphenols, nature-derived substances present in vegetation, including fruits and vegetables, have received considerable attention in various fields of science due to their probable wellness merits, particularly in the treatment and hindrance of cancer. This review focuses on the immunomodulatory effects of polyphenols in breast cancer, emphasizing their capacity to influence the reaction of adaptive and innate immune cells within the tumor-associated environment. Polyphenols are implicated in the modulation of inflammation, the enhancement of antioxidant defenses, the promotion of epigenetic modifications, and the support of immune functions. Additionally, these compounds have been shown to influence the activity of critical immune cells, including macrophages and T cells. By targeting pathways involved in immune evasion, polyphenols may augment the capacity of the defensive system to detect and eliminate tumors. The findings suggest that incorporating polyphenol-rich foods into the diet could offer a promising, collaborative (integrative) approach to classical breast cancer remedial procedures by regulating how the defense mechanism interacts with the disease.

## 1. Introduction

Breast cancer ranks among the top causes of cancer fatalities globally, with increasing incidence rates despite advancements in treatment [1,2]. This shift in focus toward prevention highlights the potential role of dietary interventions [3]. One such promising avenue is polyphenols, present in vegetable-derived products, which are associated with a myriad of health advantages, particularly in reducing cancer risk, owing to their antioxidant and inflammatory-suppressive qualities [3,4,5]. Beyond these well-established features, polyphenols can also influence the human body’s defense system and epigenetic regulation [6]. Polyphenols offer the potential for primary prevention by reducing the risk before the cancer develops [7]. They achieve this by modulating the molecular pathways associated with carcinogenesis and reversing epigenetic alterations, such as histone methylation and acetylation, DNA methylation, and microRNA expression [5,6,7,8,9].

Polyphenols, by modulating key modifications that influence tumor development, offer a potential approach for preventing breast cancer initiation and progression [10]. For instance, epigallocatechin gallate (EGCG), resveratrol, and curcumin have been thoroughly investigated for their remarkable potential in combating cancer. EGCG has been indicated to alleviate the development of breast cancer, induce apoptosis, and inhibit tumorigenesis [7,9,11,12]. Similarly, the influence of resveratrol and curcumin on epigenetic mechanisms and various signaling pathways, including those responsible for inflammation, cell survival, and metastasis implicated in the pathogenesis of breast cancer, has been extensively examined in the scientific literature [13]. Additionally, the immunomodulatory effects of polyphenols may enhance the body’s natural immune defenses, creating an environment that hinders tumor formation and progression [3,14]. In recent years, research has increasingly focused on understanding the connection between diet and cancer prevention, with particular interest in plant-derived compounds [13]. Polyphenols, among the most abundant classes of phytochemicals in human diets, have drawn significant attention due to their potential restorative impact across distinct types of cancers, including breast cancer [13]. These compounds are categorized into flavonoids, phenolic acids, lignans, and stilbenes, with notable examples being EGCG, resveratrol, curcumin, and quercetin [3,9,10,11]. The ability of these polyphenols to modulate both tumor cells and the tumor milieu, mainly through the defense system, opens new avenues for their integration into cancer therapy.

This review aims to fill a critical gap in understanding how polyphenols may contribute to breast cancer prevention through their immunomodulatory and epigenetic effects. By synthesizing available preclinical and clinical evidence, this analysis will provide insights that could inform future research and therapeutic strategies focused on dietary polyphenols as preventive agents.

## 2. Materials and Methods

Identification of multiple empirical and review articles was conducted through PubMed and Google Scholar databases. The search utilized a range of keywords, including “breast cancer”, “immunomodulatory effects of dietary polyphenols”, “dietary polyphenols”, “epigenetic effects of dietary polyphenols”, “dietary polyphenols in breast cancer”, and “breast cancer therapies”. Comprehensive searches for relevant research and review references were performed on PubMed. Descriptive statistical analyses and meta-analyses were not conducted.

## 3. Breast Cancer

**Figure 1:**
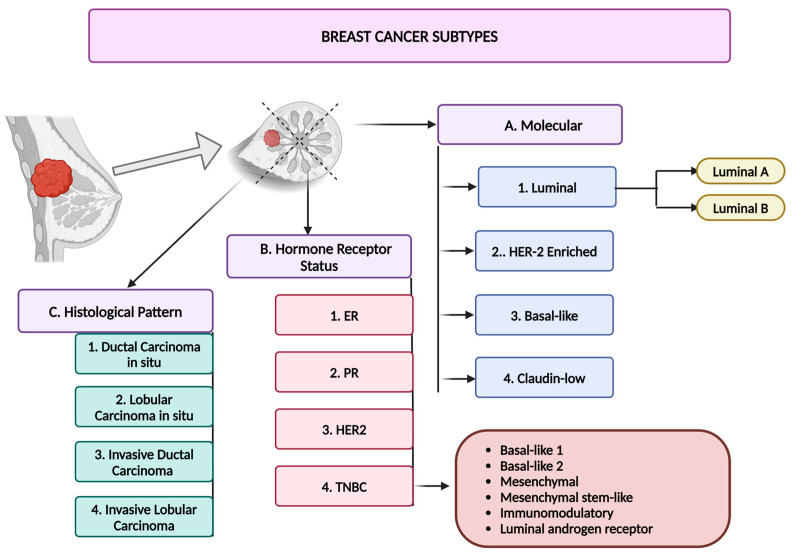
Breast cancer and its subtypes. (A) Molecular classification. (B) Hormone receptor status classification. (C) Histological classification. Created in BioRender. Lab, T. (2024) https://BioRender.com/u39p454 (accessed on 4 November 2024). HER2-enriched breast cancer carries a high risk of recurrence and neoplastic dissemination [24,25,26,27]. Approximately no more than three out of twenty breast cancer subtypes are classified as HER2 positive [24,26]. HER2-enriched breast cancers have HER2 expression while lacking ER and PR [26,27]. Basal-like breast cancer constitutes around one-fifteenth to one-fifth of all breast cancer incidences and lacks the expression of any HR [13,26,27,28]. This results in TNBC being the most aggressive and metastatic subtype, with a higher likelihood of early relapse and poor prognosis [13,27,28]. TNBC comprises six well-defined subtypes: basal-like 1 (BL1), basal-like 2 (BL2), immunomodulatory (IM), luminal androgen receptor (LAR), mesenchymal (M), and mesenchymal stem-like (MSL) [15,29,30,31,32,33]. Claudin-low breast cancer (CLBC) is distinguished by reduced tight junction proteins such as Claudins 3 and 7 and adhesion proteins [15,34,35]. CLBC represents 7–14% of all aggressive breast cancers and is connected to a low survival rate, as these tumors typically test negative for ER, PR, and HER2 [15,34,35]. The hormone receptor status can further categorize breast cancers based on hormone receptor expression (HR+), such as ER- and PR-positive breast cancers [26,28,36]. Ductal carcinoma in situ (DCIS), lobular carcinoma in situ (LCIS), Invasive Ductal Carcinoma (IDC), and Invasive Lobular Carcinoma (ILC) are histological characteristics of breast cancer subtypes [23,26,34,35,36].

Breast cancer is a widespread form of cancer in women worldwide, with both its occurrence and mortality rates recently increasing [15]. In accordance with the latest estimates released by the International Agency for Research on Cancer (IARC), the year 2022 witnessed reports of approximately 20 million new instances of cancer, along with 9.7 million fatalities [16]. Breast cancer ranked as the second predominant type of cancer, representing 11.6% of all new cancer cases globally [16]. Breast cancer can be classified into different subtypes based on molecular features, hormone receptor status, and histological patterns (Figure 1). The molecular subtypes of breast cancers are Luminal, Her2-enriched, basal-like, and Claudin-low breast cancers [17,18]. Luminal breast cancers account for 60–70% of all breast cancer types in economically advanced countries [19]. Luminal breast cancer is divided into two subtypes: Luminal A, which has a slower growth rate and better prognosis, and Luminal B, which has a faster growth rate and worse prognosis [15,20,21,22]. Luminal A breast cancers are characterized by the absence of HER2 and the presence of either an ER or a PR [23]. These cancers typically manifest in the mammary duct epithelium. In comparison, Luminal B breast cancers are estrogen receptor positive and may be PR negative and/or HER2 positive [15,17,20,22].

## 4. Immune System Regulation in Breast Cancer

**Figure 2:**
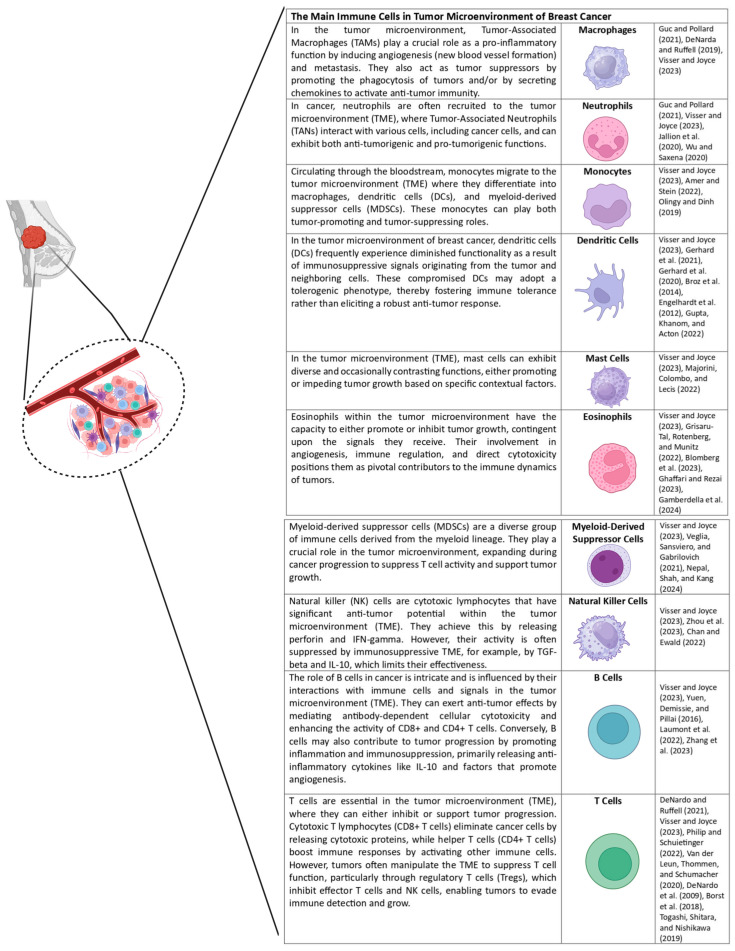
The diversity of immune system cells in the cancer-associated environment of breast cancer [43,44,45,46,47,48,49,50,51,52,53,54,55,56,57,58,59,60,61,62,63,64,65,66,67,68,69,70,71]. Created in BioRender. Lab, T. (2024) https://BioRender.com/s56n309 (accessed on 4 November 2024).

The immune system is a sophisticated network that aids the body in defending against diseases. The management of the defense mechanism in breast cancer is also a complicated progression impacted by the defensive system’s cells and cancer cells and the surrounding microenvironment, which encompasses cancer and stromal cells, an extracellular matrix (ECM), signaling molecules, blood vessels, and hypoxic regions [26,37,38,39]. Cancer-associated fibroblasts are the primary cell type in the microenvironment [37,38,40]. In addition, it comprises a diverse array of immune cells (Figure 2), including lymphocytes, macrophages, myeloid-derived stromal cells, and cytokines [37,41,42].

## 5. Breast Cancer Treatment Types

Breast cancer (BC) treatment necessitates a multidisciplinary approach due to its various subtypes. There are numerous conventional and innovative treatment methods available, including surgery, radiation therapy, chemotherapy, hormone therapy, and immunotherapies [72]. These treatments can be used individually or in combination to address breast cancer. For early-stage invasive breast cancer, surgery and mastectomy are often preferred as they boast an 85–95% success rate in preventing tumor recurrence [72,73]. Radiotherapy plays a crucial role in the comprehensive treatment of breast cancer, and it is frequently utilized in conjunction with surgical intervention and chemotherapy to diminish the likelihood of disease recurrence, particularly subsequent to breast-conserving surgery or mastectomy [74,75]. Chemotherapy is a standard treatment method for breast cancer, incorporating the administration of drugs to eliminate cancer cells [72,76]. Its applicability across various stages of breast cancer underscores its diverse therapeutic purposes, tailored to individual cases, and it reduces the risk of recurrence of breast cancer to only 30% [72,76,77]. Hormone therapy serves as a fundamental treatment for hormone receptor-positive breast cancers, where cancer cells proliferate in response to estrogen or progesterone [78,79,80]. This therapeutic approach operates by either obstructing these hormones or diminishing their levels to decelerate or halt cancer progression [79,80,81]. Immunotherapy has garnered significant attention due to its potential to harness the body’s defense system to develop innovative therapeutic strategies [82,83]. Adoptive cell therapies (e.g., CAR-T cell therapy), vaccines (e.g., Personalized Peptide Vaccine), cytokine therapies (e.g., anti-IL-1β), monoclonal antibodies (e.g., Herceptin and Pertuzumab), and immune checkpoint (e.g., PD-1 and CTLA4) blockade represent foundational forms of immunotherapy [72,82,83,84,85,86,87,88,89].

Different breast cancer treatment modalities have distinct advantages and limitations depending on the breast cancer subtype. However, immunotherapy has been favored over traditional therapies in recent years for several reasons. Unlike chemotherapy or radiotherapy, which targets cancerous and healthy cells, immunotherapy harnesses the body’s immune system to selectively attack cancer cells, resulting in fewer side effects overall [84,90]. Although immunotherapy may have defense system-related side effects, they are generally less severe than those associated with chemotherapy or radiation [89,90]. Immunotherapy has also shown significant success in treating aggressive forms of breast cancer, such as TNBC, which is typically less responsive to hormonal or targeted therapies [91]. The efficacy of immunotherapy in the treatment of various subtypes of TNBC is known to vary, highlighting the ongoing need for safe and effective methods for both prevention and treatment [90,92,93,94,95]. This challenge has sparked growing interest in complementary approaches, such as dietary interventions rich in polyphenols, to enhance treatment outcomes [94,95].

## 6. Polyphenols and Their Importance

**Figure 3:**
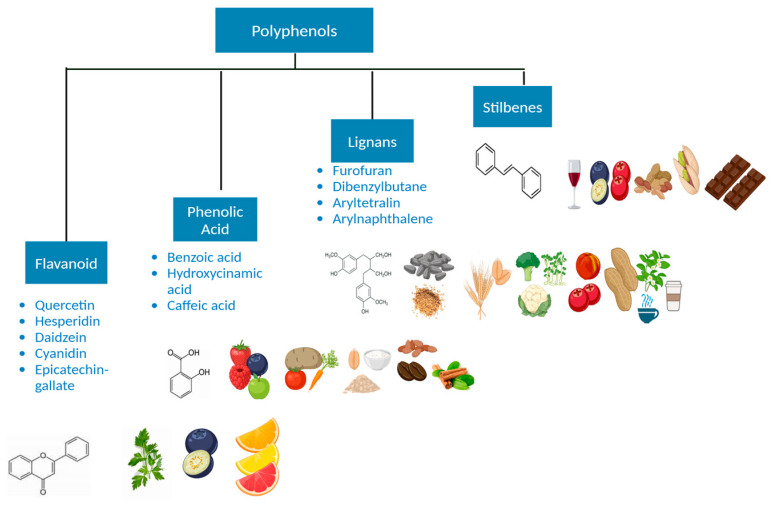
General subtypes of polyphenols. Created in BioRender. Lab, T. (2024) https://BioRender.com/q91l231 (accessed on 4 November 2024).

Polyphenols represent a wide range of natural substances found in plant-derived foods. They are known for their antioxidant impacts and have garnered significant attention due to their potential health advantages [96,97,98]. Structurally, polyphenols contain multiple phenol units, which play a key role in their biological activity [96,99]. These compounds are abundant in fruits, vegetables, tea, coffee, wine, spices, and whole grains [99]. Due to their wide availability in plant-based diets, polyphenols have become a central focus in nutritional research, particularly concerning disease prevention and health promotion [99]. Polyphenols (Figure 3) can be classified into four categories according to their chemical structure.

Flavonoids: This is the largest group of polyphenols, accounting for over half of all dietary polyphenols. The basic framework is made up of two aromatic rings connected by three carbons. Berries, citrus, oranges, onions, tea, and red wines are all rich in flavonoids [100,101]. Subclasses of flavonoids include flavanols (e.g., quercetin and catechins in green tea) and anthocyanins (e.g., pigments in berries) [102].

Phenolic Acids: These compounds contain a benzene ring with hydroxyl and carboxyl groups and are prevalent in foods like coffee, whole grains, and certain fruits [103]. They include caffeic acid and ferulic acid, both famous for their anti-cancer and inflammatory properties [103].

Stilbenes: While less common than flavonoids with two interconnected benzene rings, stilbenes such as resveratrol (found in grapes, red wine, and berries) are highlighted for the potential significance of their role in promoting longevity and cancer prevention [104,105].

Lignans: Found in seeds, particularly flaxseeds, as well as whole grains and vegetables, lignans are phytoestrogens that are believed to contribute to hormone-related cancer prevention [106].

## 7. Dietary Polyphenols and Their Impact on Immune System

Dietary polyphenols have been shown to impact multiple aspects of immune function, providing a means to restore or enhance anti-tumor immunity [107,108,109]. For instance, EGCG, a green tea polyphenol, is highlighted for its ability to constrain the rapid growth of cancer cells and promote apoptosis [110]. Beyond its direct effects on cancer cells, EGCG modulates immune responses by boosting the activity of cytotoxic T cells and natural killer cells, key players in targeting and killing cancer cells [109,111,112]. Additionally, EGCG has been shown to downregulate immunosuppressive regulatory T cells (Tregs) and myeloid-derived suppressor cells (MDSCs) within the tumor microenvironment, thereby restoring an environment conducive to immune-mediated tumor destruction [112,113].

Curcumin, another well-known polyphenol from turmeric, exerts immunomodulatory effects by targeting a variety of immune pathways [112,114]. It can inhibit inflammation-stimulating immune modulators such as IL-6 and TNF-α, which are often elevated in patients with cancer and contribute to a chronic inflammatory state that promotes tumor growth [112,115,116]. Curcumin also elevates NK and dendritic cells’ activity, facilitating a more robust anti-tumor immune response. Furthermore, its ability to modulate immune checkpoints, such as programmed cell death protein 1 (PD-1), positions it as a favorable phytochemical for potential synergistic application in conjunction with immune checkpoint inhibitors in treating breast cancer [117,118].

Resveratrol, found in grapes and red wine, has also shown significant immunomodulatory potential. It has been reported to suppress the expression of programmed death-ligand 1 (PD-L1) in breast cancer cells, thereby reducing T cell exhaustion and promoting protection against tumor formation [119]. Resveratrol also influences the polarization of TAMs from a tumor-promoting M2 phenotype to a tumoricidal M1 phenotype, creating a more hostile environment for breast cancer cells [120,121].

The anti-inflammatory effects of quercetin, a flavonoid found in many fruits and vegetables, contribute to its immunomodulatory potential in breast cancer [122]. Quercetin can inhibit the formation of immune mediators that induce inflammation, like IL-1β and IL-8, which contribute to a tumor-promoting inflammatory environment [123]. By reducing this inflammation, quercetin may help to tip the balance in favor of an anti-tumor immune response [124].

## 8. Role of Polyphenols in Regulating the Immune System

Polyphenols are well known for their antioxidant properties, but their role in immune system regulation has gained increasing attention in recent years [3,125]. Polyphenols modulate immune responses through several mechanisms, affecting diverse aspects of immune function by interacting with specific receptors on various defense system cells [3,109]. Their immunomodulatory properties can contribute to the prevention and treatment of a range of diseases, including breast cancer [126].

### 8.1. Modulation of Immune Cells

Polyphenols exert their effects by influencing assorted defensive cells, such as T cells, B cells, NK cells, macrophages, and dendritic cells [3]. These immune cells possess specific recognition units that interact with polyphenols. For example, EGCG binds to the ZAP-70 receptor, whereas resveratrol links to the Sp1 receptor on different immune cell types. This receptor–ligand correspondence initiates signaling cascades that promote apoptosis and immune responses, such as increasing the activity of cytotoxic T cells and NK cells [3,121,127,128,129]. In addition, polyphenols help balance the immune system by stimulating the diversification of M1 macrophages (pro-inflammatory and anti-tumor) over M2 macrophages (associated with tumor growth and immune suppression) [130]. This shift in macrophage activity can enhance immunological monitoring and the body’s capacity to fight tumors.

### 8.2. Regulation of Inflammatory Responses

Chronic inflammation is a major driver of many diseases, including cancer [131]. Polyphenols play a crucial role in modulating the inflammatory response, primarily by affecting the production of cytokines—small signaling proteins that regulate immune and inflammatory responses [128]. Polyphenols like curcumin (from turmeric) and quercetin (from fruits and vegetables) reduce the secretion of cytokines driving inflammation, such as TNF-α, IL-6, and IL-1β, related to prolonged inflammation response and tumor progression [132,133].

At the same time, polyphenols promote the generation of interleukin-10, an inflammatory mediator, which helps mitigate excessive immune responses and prevent tissue damage [134,135]. This ability to regulate the balance of inflammatory driving and prevent cytokines helps maintain immune homeostasis and prevents the immune system from becoming more activated, which can lead to autoimmune disorders or chronic inflammation [3,135].

### 8.3. Immune Checkpoint Regulation

Cancer cells often evade detection and elimination by the immune system by exploiting immune checkpoints, which are regulatory molecules expressed on immune cells that need to be activated or deactivated to start an immune response [136,137]. Polyphenols, such as genistein (from soy) and resveratrol, govern these immune checkpoints [136]. For example, they can inhibit the PD-1/PD-L1 pathway, a mechanism through which cancerous cells subdue T cell activity, thereby helping the body’s defense system recognize and counteract tumor cells more effectively [62].

PD-1 is a transmembrane binding site on immune cells, including helper and cytotoxic T cells and NK cells [138,139,140,141]. The expression of PD-1 on T cells is governed by several pathways, including HIF-1α, NF-κB, PI3K/AKT/mTOR, and JAK/STAT [136,139,142]. Its ligand, PD-L1, is often overproduced in cancer cells, facilitating immune evasion [143]. The presence of PD-L1 in tumors is governed by the MAPK pathway, HIF-1α, and epigenetic mechanisms such as histone deacetylation, along with the JAK/STAT, Wnt, and PI3K/AKT/mTOR pathways [136,139]. Polyphenols have the potential to inhibit these pathways, reducing PD-L1 levels in cancer cells and enhancing T cell activation, which improves the recognition of cancer cells.

### 8.4. Regulation of T Cells and B Cells

Polyphenols exert significant impacts on the adaptive defense system by modulating lymphocytes’ activity. They are known to enhance the proliferation of B and T cells while concurrently suppressing the function of regulatory T cells and specific subsets of T helper cells, including Th9 and Th17 [3]. For example, resveratrol and curcumin can promote a Th1-type response, which is essential for anti-tumor immunity, while reducing the overactivation of Th2-type responses associated with allergic reactions and immune suppression [144]. Polyphenols also aid in the production of antibodies that are crucial for targeting pathogens and cancer cells by enhancing B cell function [111].

## 9. Synergistic Potential with Conventional Therapies

The immunomodulatory properties of dietary polyphenols suggest that they could be used in conjunction with conventional cancer therapies [125,145,146,147,148]. Chemotherapy and radiotherapy, while effective at killing tumor cells, often induce immunosuppressive effects, which can limit their long-term efficacy [149]. Polyphenols have the potential to enhance immune function, which could help mitigate the adverse effects associated with various conditions. Although conventional treatments can also impact healthy cells, polyphenols demonstrate the capacity to improve these outcomes due to their antioxidant properties, thereby reducing the side effects that traditional therapies may impose on the defense system. Furthermore, the integration of polyphenols with immunotherapeutic approaches, such as immune checkpoint inhibitors, may yield a synergistic effect by augmenting the activity of immune cells and overcoming the immune resistance mechanisms utilized by breast cancer cells [150,151].

## 10. Epigenetic Regulation by Polyphenols in Cancer Immunomodulation

Beyond their direct interactions with immune cells, polyphenols can modulate immune responses at the epigenetic level [111,152,153]. Epigenetic modifications, including histone methylation, acetylation, and DNA methylation, are pivotal in regulating gene expression [152,154,155]. Aberrant epigenetic changes are a hallmark of cancer, including in breast cancer, where they can silence tumor suppressor genes and activate oncogenes [9]. Dietary polyphenols have been shown to reverse these modifications, thereby restoring normal cellular functions and immune responses [156,157,158].

For example, EGCG has been reported to inhibit DNA methyltransferases (DNMTs), enzymes that add methyl groups to DNA, and silence gene expression [159,160]. By inhibiting DNMTs, EGCG can reactivate tumor suppressor genes and advocate the immune system’s ability to dispose of breast cancer cells [160,161]. Similarly, curcumin has been shown to modulate histone acetylation, thereby facilitating the reactivation of immune-related genes that had been silenced in the tumor microenvironment. Furthermore, curcumin influences the PD-1/PD-L1 immune checkpoint [9,162]. Moreover, resveratrol has been demonstrated to inhibit histone acetylation while regulating microRNAs, enhancing T cell activation, and reducing tumor progression. Quercetin monitors histone acetylation and modulates the subtype switching of macrophages.

## 11. Specific Polyphenols Used in Breast Cancer Treatment

As shown in Table 1, EGCG, resveratrol, curcumin, quercetin, genistein, apigenin, and pterostilbene are commonly researched polyphenols for the primary prevention and management of breast cancer [163,164,165,166,167,168,169,170,171,172,173,174,175,176,177,178,179,180,181,182,183,184,185,186,187,188,189,190,191,192].

### 11.1. Epigallocatechin-3-Gallate (EGCG)

**Figure 4:**
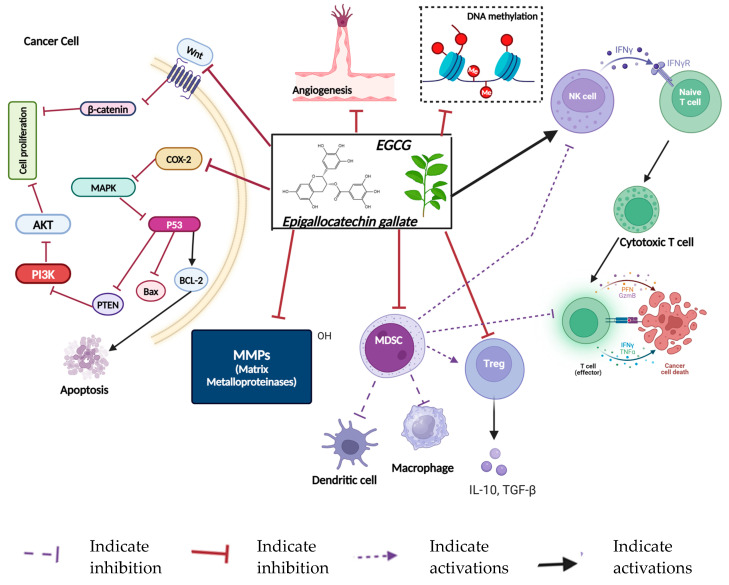
The common impacts of EGCG (epigallocatechin gallate) in breast cancer. EGCG influences multiple pathways, reducing cell proliferation and promoting apoptosis by inhibiting the PI3K/AKT pathway and activating tumor suppressor proteins like p53, leading to Bax activation and BCL-2 suppression. EGCG blocks the Wnt/β-catenin and MAPK pathways, reducing COX-2 levels and consequently lowering inflammation. It also suppresses matrix metalloproteinases (MMPs), preventing extracellular matrix degradation and angiogenesis. Created in BioRender. Lab, T. (2024) https://BioRender.com/e47a401 (accessed on 4 November 2024).

EGCG (Figure 4) is a natural polyphenol, the most abundant catechin in green tea, and has been extensively studied for its anti-cancer properties, particularly in breast cancer. Its effects on cancer cells include inhibiting cell proliferation, inducing apoptosis, and modulating multiple signaling pathways, such as PI3K/Akt and MAPK [193]. EGCG has been shown to inhibit EGFR or ErBb1 and 2, which are often found to be overexpressed in breast cancer [193]. EGCG has also been shown to inhibit the expression of matrix metalloproteinases (MMPs), which play a crucial role in the progression and dissemination of tumors [194]. Furthermore, it exhibits anti-angiogenic properties by downregulating VEGF expression, thereby repressing new blood vessel generation that supplies tumors [195]. Preclinical studies have demonstrated that EGCG sensitizes breast cancer cells to conventional therapies, such as tamoxifen and trastuzumab, suggesting its potential as an adjunctive treatment [196,197].

### 11.2. Resveratrol

**Figure 5:**
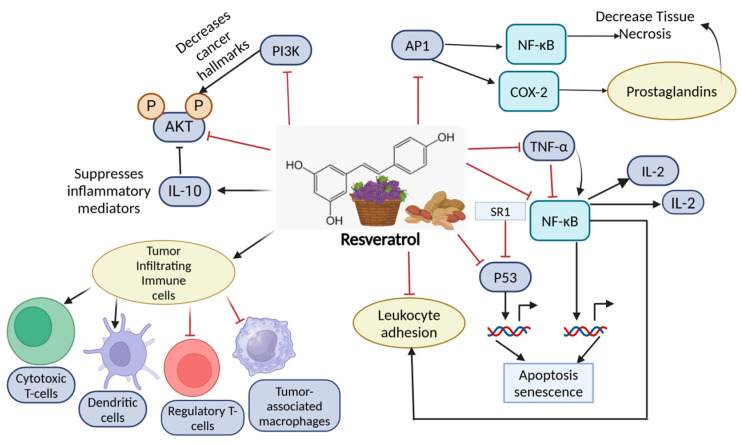
The immunomodulatory effects of resveratrol on cancer and inflammation. Resveratrol impacts several signaling pathways and immune cells, contributing to anti-cancer and anti-inflammatory actions. Resveratrol alters cancer hallmarks by suppressing inflammatory mediators while promoting IL-10 production through the inhibition of the PI3K/AKT pathway. This influences tumor-infiltrating immune cells, such as cytotoxic T cells, dendritic cells, Tregs, and tumor-associated macrophages, enhancing immune response against tumors. It reduces TNF-α, impacting IL-2 and IL-10 levels, thus modifying immune and inflammatory responses within the tumor microenvironment. Created in BioRender. Lab, T. (2024) https://BioRender.com/e44e698 (accessed on 4 November 2024).

Resveratrol (Figure 5), a stilbene found in grapes, berries, and peanuts, has gained significant attention for its chemo-preventive and healing impacts in resistance to breast cancer [149,198]. The mechanisms of resveratrol include inducing programmed cell death, inhibiting cell proliferation, and suppressing the growth of estrogen receptor (ER)-positive breast cancer by downregulating ERα signaling [199]. Resveratrol has been shown to inhibit metastasis in TNBC cell lines by altering the TGF-β1-induced epithelial matrix transformation, thus providing promising primary prevention options for these types of cancers that have no cure except for chemotherapies. Moreover, resveratrol influences multiple molecular pathways, including the NF-κB and STAT3 pathways, which are crucial for cancer cell survival and proliferation [200,201]. In TNBC, resveratrol has shown the ability to reduce tumor growth by modulating the expression of oncogenic microRNAs and increasing tumor suppressor genes. Its capacity to penetrate the blood–brain barrier hints at its potential to help prevent the spread of breast cancer to the brain, which is a frequent issue in the advanced stages of the disease [202].

### 11.3. Curcumin

**Figure 6:**
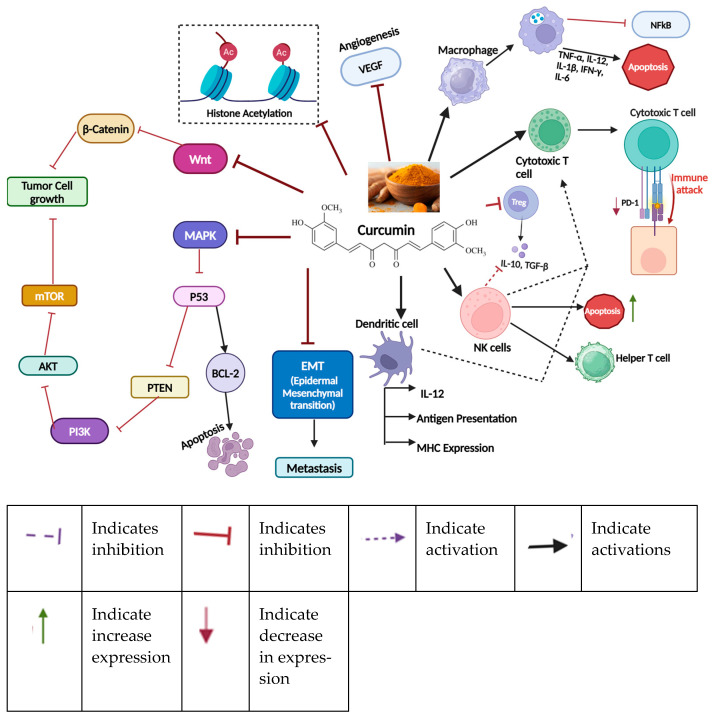
Important effects of curcumin on breast cancer. Curcumin suppresses Wnt and MAPK pathways, along with epithelial–mesenchymal transition (EMT) in the tumor microenvironment, histone acetylation, angiogenesis, and T-regulatory cells, which play significant roles in cancer development. In contrast, curcumin activates dendritic cells, which promotes the secretion of IL-12 and activates cytotoxic T cells. Furthermore, curcumin stimulates macrophages, cytotoxic T cells, and natural killer (NK) cells, leading to the activation of T helper cells and the increased apoptosis of tumor cells. Notably, curcumin also reduces PD-1 expression on T cells, thereby enhancing the immune response against tumor cells. Created in BioRender. Lab, T. (2024) https://BioRender.com/w77n343 (accessed on 4 November 2024).

Curcumin, a key polyphenol (Figure 6) found in turmeric (*Curcuma longa*), is well recognized for its anti-inflammatory and anti-cancer properties. In breast cancer, it targets multiple signaling pathways, such as PI3K/Akt/mTOR and Wnt/β-catenin, which play pivotal roles in tumor development and progression [203,204]. Curcumin promotes apoptosis in cancer cells by activating caspase enzymes and downregulating anti-apoptotic proteins like Bcl-2 and Bcl-xL [205]. Moreover, it inhibits the epithelial–mesenchymal transition (EMT), a critical process involved in cancer metastasis [206]. Its anti-inflammatory effects are particularly relevant in breast cancer as chronic inflammation is strongly linked to tumor growth [207]. Additionally, curcumin enhances the effectiveness of chemotherapy and helps combat drug resistance in breast cancer cells [207,208].

### 11.4. Quercetin

**Figure 7:**
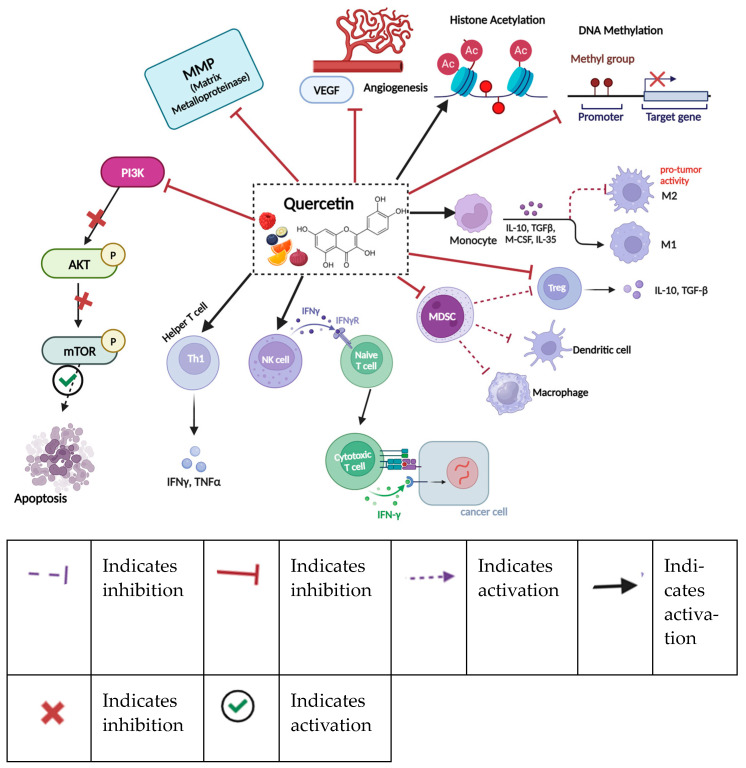
Diverse effects of quercetin on breast cancer. Quercetin effectively inhibits the PI3K pathway, angiogenesis, DNA methylation, and the activity of myeloid-derived suppressor cells (MDSCs), which typically suppress the functions of dendritic cells (DCs), macrophages, and T-regulatory cells. Additionally, quercetin promotes the secretion of IL-10 and enhances histone acetylation. It also activates T helper cells, natural killer (NK) cells, and monocytes, contributing to a more robust immune response. Created in BioRender. Lab, T. (2024) https://BioRender.com/i98p170 (accessed on 4 November 2024).

Quercetin, a flavonoid (Figure 7) abundantly present in apples, onions, and citrus fruits, exhibits potent oncolytic outcomes in breast cancer via multiple molecular and cellular mechanisms [209]. It promotes apoptosis in breast cancer cells by upregulating pro-apoptotic proteins such as Bax and activating caspases while simultaneously reducing the levels of apoptosis-preventing proteins like Bcl-2 [210]. Beyond its direct anti-cancer activity, quercetin significantly influences immune modulation, contributing to its tumor-suppressive properties [3,211]. It enhances the immune system’s capacity to target and eliminate cancer cells by activating cytotoxic T cells and regulating T-helper cell responses to ensure immune homeostasis. Quercetin also modulates macrophage activity, suppressing the pro-inflammatory M1 phenotype and reducing the secretion of inflammatory cytokines such as TNF-α and IL-6, which are frequently elevated in tumor microenvironments [212]. Concurrently, it promotes a shift towards the M2 macrophage phenotype, fostering tissue repair and anti-inflammatory effects [133,213]. Furthermore, quercetin enhances the cytotoxic function of natural killer (NK) cells, strengthening their ability to directly target and destroy tumor cells [214]. The dual role of quercetin in immune regulation and tumor suppression underscores its potential as a multifaceted agent for the prevention and treatment of breast cancer.

### 11.5. Genistein

Genistein, a prominent soy-derived isoflavone, has gained considerable attention for its role in breast cancer prevention and treatment [215]. Studies suggest that Asian women consuming soy-rich diets have a lower incidence or occurrence of breast cancer [216]. In ER-positive breast cancer, genistein inhibits cell proliferation by competing with endogenous estrogens for ER binding, thereby reducing estrogen-driven tumor growth [217]. Additionally, genistein inhibits tyrosine kinase activity, affecting key signaling pathways such as PI3K/Akt and MAPK. This includes suppressing protein levels like MEK5 (mitogen-activated protein kinase 5) and ERK5 (extracellular signal-regulated kinase 5), [218,219,220], which are essential for cancer cell survival, consistent with cell growth inhibition and the induction of apoptosis [221,222]. Genistein induces apoptosis primarily through caspase activation, the modulation of endoplasmic reticulum stress regulators, and an increase in the Bax/Bcl-2 ratio. Other proposed mechanisms include proteasome activity inhibition, the downregulation of the anti-apoptotic protein survivin, and the suppression of angiogenesis and tumor progression in breast cancer [223]. Finally, studies also indicate that genistein enhances the efficacy of chemotherapeutic agents like tamoxifen, making it a potential adjunctive therapy in breast cancer treatment [224].

### 11.6. Apigenin

Apigenin, a flavone present in parsley, celery, and chamomile, has demonstrated promising anti-cancer properties in breast cancer research [225]. It exerts its effects by inducing cell cycle arrest at the G2/M phase and triggering apoptosis through caspase-3 activation while downregulating anti-apoptotic proteins such as Bcl-2 [226]. Beyond its pro-apoptotic activity, apigenin inhibits metastasis by reducing the expression of matrix metalloproteinases (MMPs) and suppressing epithelial–mesenchymal transition (EMT) [227,228]. Additionally, it mitigates inflammation within the tumor microenvironment by inhibiting the NF-κB pathway, a critical regulator of inflammation-driven tumor progression [229,230]. Notably, apigenin enhances the vulnerability of breast cancer cells to radiation and chemotherapy, highlighting its feasibility as a complementary therapeutic agent [231].

### 11.7. Pterostilbene

Pterostilbene, a naturally occurring demethylated derivative of resveratrol, is found in blueberries and grapes. It has shown potential in breast cancer therapy through its strong cancer-preventive features, including the inhibition of cell proliferation, the induction of apoptosis, and the suppression of metastasis [232,233]. Notably, pterostilbene influences key molecular pathways such as PI3K/Akt and JAK/STAT, which are essential for the survival and growth of cancer cells [234]. In triple-negative breast cancer (TNBC) models, pterostilbene has been shown to impede tumor growth and metastasis by reducing the expression of oncogenic signaling molecules, including MMPs and VEGF [235,236]. Furthermore, its antioxidant properties alleviate oxidative stress in cancer cells, amplifying its therapeutic potential [192].

## 12. Discussion

Polyphenols constitute an assortment of naturally occurring nutrients common in plant-based foods and have attracted considerable interest due to their multifaceted bioactive properties [3,237]. These compounds are recognized for their antioxidant capabilities, anti-inflammatory effects, the modulation of estrogen activity, the inhibition of carcinogenic pathways, epigenetic modifications, and their roles in inhibiting angiogenesis and cellular migration [3,5,97,238,239,240,241]. Additionally, polyphenols function as immunomodulators and are readily accessible through dietary sources [12,98,242,243,244].

The potential of polyphenols as a primary preventive strategy for breast cancer is particularly noteworthy given their immunomodulatory effects. Breast cancer is regarded as one of the less immune-inducing cancer types; thus, enhancing the immune response within the tumor microenvironment may be advantageous for preventing disease onset [241,242]. Based on our comprehensive review of the existing literature, polyphenols may serve as promising therapeutic agents for the treatment of various subtypes of breast cancer.

Empirical studies indicate that polyphenols can modulate the activity of several immune cell types within the breast cancer tumor microenvironment [134,145]. For instance, epigallocatechin gallate (EGCG) has been demonstrated to activate natural killer (NK) cells and induce cytotoxic T cells, thereby promoting anti-tumor activity [109,111,112]. Similarly, pterostilbene has been shown to facilitate the transition of macrophages from the pro-inflammatory M1 phenotype to the anti-inflammatory M2 phenotype [245]. This phenotypic shift is instrumental in mitigating chronic inflammation and fostering tissue repair, resulting in a more balanced immune response that is less susceptible to excessive inflammatory damage. Moreover, polyphenols generally downregulate regulatory T cell (Treg) expression within the tumor microenvironment, thereby counteracting immune suppression [3]. Cancer cells frequently evade immune surveillance through mechanisms that involve checkpoint inhibitors such as PD-1 [137]. However, polyphenols, including genistein (derived from soy) and resveratrol, have been found to modulate these immune checkpoints by inhibiting the PD-1/PD-L1 pathway [136,139,246]. This modulation enhances the immune system’s ability to recognize and effectively target tumor cells.

Current therapeutic modalities for breast cancer, including immunotherapy and chemotherapy, primarily focus on the treatment or reduction in cancerous cells. These approaches often lead to numerous undesirable outcomes, such as significant autoimmune reactions impacting the skin, liver, lungs, and other organs [137,247]. Additionally, the high costs associated with immunotherapy and the requirement for frequent clinical administration constrain its applicability as a widespread preventive measure. Notably, these conventional treatments do not emphasize cancer prevention at the initial stages. In contrast, polyphenols are naturally present in various dietary sources and can be consumed regularly without necessitating medical supervision [248]. The long-term intake of polyphenol-rich foods has been linked to minimal side effects, thus rendering them suitable for strategies aimed at the primary prevention of breast cancer [249].

## 13. Conclusions

To enhance the effectiveness of immunotherapy and polyphenols for specific breast cancer subtypes, particularly triple-negative breast cancer (TNBC), researchers are examining combination treatments that pair immunotherapy drugs with polyphenols or integrate various polyphenolic compounds. However, polyphenols can interact with certain medications and foods due to their distinct composition, potentially leading to adverse effects [134]. Moreover, their low absorption and fast elimination from the body present challenges for their use as therapeutic agents in clinical practice, underscoring the need for further research in this area [98,134]. Future investigations should continue to explore how polyphenols contribute to cancer prevention and their potential synergistic effects alongside other preventive strategies, such as lifestyle modifications and targeted therapies [125,145,146,147,148].

## Figures and Tables

**Table 1:** Common polyphenols used in breast cancer treatment.

Name	Their Importance	Epigenetic Effects	Immunomodulatory Effects	Found In
Epigallocatechin gallate (EGCG)	Strong antioxidant and anti-inflammatory properties and potential anti-cancer properties; inhibition of tumor cell proliferation [163,164]	DNA methylation inhibition, histone modification, miRNA expression changes [165]	It enhances cytotoxic T cell function, inhibits Tregs, and increases macrophages. It also reduces inflammation and suppresses NF-kB signaling [166].	Green tea, white tea [167] 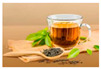
Resveratrol	Cardioprotective and anti-inflammatory properties, potential cancer prevention; promotes cell death in cancer cells [168,169]	Promotes histone acetylation, inhibits DNA methyltransferases, modifies miRNA [170,171]	It modulates T helper cell activity, particularly by promoting a shift from a pro-inflammatory Th1 response (which could contribute to chronic inflammation) to a more balanced Th2 or regulatory T cell (Treg) response, reducing inflammation while maintaining immune surveillance against cancer [172].	Grapes, red wine, berries, peanuts [173] 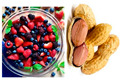
Curcumin	Anti-inflammatory and antioxidant properties; modulates multiple cell signaling pathways; potential anti-cancer agent [174]	Histone acetylation inhibition [175,176]	It modulates the differentiation of T helper cells, reduces the expression of immune suppressive Tregs, and modulates macrophages and NK cells [177].	Turmeric (*Curcuma longa*) [178] 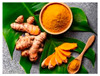
Quercetin	Anti-inflammatory, antioxidant, and antiviral properties; may enhance immunity and reduce cancer risk [179]	Demethylation of tumor suppressor genes and histone acetylation, modifies miRNA [180]	Monocyte activation leads to the synthesis of interleukin-10 and TGF-β, inducing M1 macrophages while inhibiting M2 macrophages, which are associated with pro-tumor activity [181].	Apples, onions, berries, leafy greens [179] 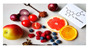
Genistein	Modulates estrogen receptors; potentially prevents breast cancer; antioxidant properties [182]	Inhibits DNA methylation, induces histone acetylation, and alters miRNA expression [183]	It enhances the activity of cytotoxic T cells. It modulates the function of T helper cells and leads to the activation of B cells and macrophages [184].	Soybeans, tofu, soy products [185] 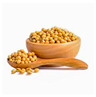
Apigenin	Anti-inflammatory and antioxidant properties; may induce apoptosis in cancer cells; neuroprotective effects [186]	Inhibits histone deacetylases, promotes DNA demethylation [187]	Inducing NK cell activity, boosting their ability to destroy tumor cells [188].	Parsley, chamomile, celery, citrus fruits [186,189] 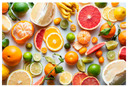
Pterostilbene	Antioxidant and anti-inflammatory properties; potential anti-cancer properties; modulates lipid metabolism [190]	Modulates histone acetylation and DNA methylation, affects miRNA profiles [191]	It suppresses macrophage activity and promotes dendritic cells’ maturation and has an antigen-presenting function [192].	Blueberries, grapes, heartwood of *Pterocarpus marsupium* [190] 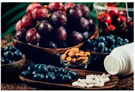

## Abbreviations

Akt	Protein Kinase B
Bax	BCL2-Associated X (Apoptosis Regulator)
BC	Breast Cancer
Bcl-2	B Cell Lymphoma Protein 2
Bcl-xl	B Cell Lymphoma-Extra Large
BL1	Basal-Like 1
BL2	Basal-Like 2
CAFs	Cancer-Associated Fibroblasts
CAR-T	Chimeric Antigen Receptor T cells
CDK4	Cyclin-Dependent Kinase 4
CLBC	Claudin-Low Breast Cancer
CTLs	Cytotoxic T lymphocytes
CTLA4	Cytotoxic T Lymphocyte-Associated Protein 4
DC	Dendritic Cells
DCIS	Ductal Carcinoma In Situ
ECM	Extracellular Matrix
EGCG	Epigallocatechin Gallate
ER	Estrogen Receptor
ER+	Estrogen Receptor-Positive
EMT	Epithelial–Mesenchymal Transition
HER2	Human Epidermal Growth Factor 2
HER2+	Human Epidermal Growth Factor 2-Positive
HR	Hormone Receptor
HR+	Hormone Receptor-Positive
HIF-1α	Hypoxia-Inducible Factor 1-Alpha
JAK/STAT	Janus Kinase–Signal Transducer and Activator of Transcription
IDC	Invasive Ductal Carcinoma
ILC	Invasive Lobular Carcinoma
IM	Immunomodulatory
LAR	Luminal Androgen Receptor
LCIS	Lobular Carcinoma In Situ
M	Mesenchymal
M1	M1-Type Macrophage
M2	M2-Type Macrophage
MAPK	Mitogen-Activated Protein Kinase
MDSCs	Myeloid-Derived Suppressor Cells
MEK5	Mitogen-Activated Protein Kinase 5
MSL	Mesenchymal Stem-Like
MMP	Matrix Metalloproteinase
mTOR	Mammalian Target of Rapamycin
NFκB	Nuclear Factor Kappa B
NK Cells	Natural Killer Cells
PD-1	Programmed Cell Death Protein 1
PD-L1	Programmed Death-Ligand 1
PI3K	Phosphoinositide 3-Kinase
PR	Progesterone Receptor
PR+	Progesterone Receptor-Positive
Sp1	Transcription Factor-Specific Protein 1
STAT3	Signal Transducer and Activator of Transcription 3
TAMs	Tumor-Associated Macrophages
Th1	Type 1 T Helper
Th2	Type 2 T Helper
Th9	Type 9 T Helper
Th17	Type 17 T Helper
TME	Tumor Microenvironment
TNBC	Triple-Negative Breast Cancer
Tregs	Regulatory T Cells
VEGF	Vascular Endothelial Growth Factor
ZAP-70	Zeta Chain-Associated 70kDa Protein Receptor
